# Advances in the application of CRISPR-Cas technology in rapid detection of pathogen nucleic acid

**DOI:** 10.3389/fmolb.2023.1260883

**Published:** 2023-09-21

**Authors:** Xiaoping Li, Jiaye Zhong, Haoyu Li, Yinbiao Qiao, Xiaolei Mao, Huayan Fan, Yiwu Zhong, Saber Imani, Shusen Zheng, Jianhui Li

**Affiliations:** ^1^ Key Laboratory of Pollution Exposure and Health Intervention of Zhejiang Province, Shulan International Medical College, Zhejiang Shuren University, Hangzhou, China; ^2^ Faculty of Medicine, Macau University of Science and Technology, Avenida Wai Long Taipa, Macau, China; ^3^ Department of Hepatobiliary and Pancreatic Surgery, Department of Liver Transplantation, Shulan (Hangzhou) Hospital, Zhejiang Shuren University School of Medicine, Hangzhou, China; ^4^ NHC Key Laboratory of Combined Multi-Organ Transplantation, Hangzhou, China; ^5^ The Organ Repair and Regeneration Medicine Institute of Hangzhou, Hangzhou, China; ^6^ Jinan Microecological Biomedicine Shandong Laboratory, Jinan, China; ^7^ Zhejiang Chinese Medical University, Hangzhou, China

**Keywords:** CRISPR-Cas, Cas9, Cas12, Cas13, Cas14, pathogen nucleic acid, rapid detection

## Abstract

*Clustered regularly interspaced short palindromic repeats* (CRISPR) and *CRISPR-associated proteins* (Cas) are widely used as gene editing tools in biology, microbiology, and other fields. CRISPR is composed of highly conserved repetitive sequences and spacer sequences in tandem. The spacer sequence has homology with foreign nucleic acids such as viruses and plasmids; Cas effector proteins have endonucleases, and become a hotspot in the field of molecular diagnosis because they recognize and cut specific DNA or RNA sequences. Researchers have developed many diagnostic platforms with high sensitivity, high specificity, and low cost by using Cas proteins (Cas9, Cas12, Cas13, Cas14, etc.) in combination with signal amplification and transformation technologies (fluorescence method, lateral flow technology, etc.), providing a new way for rapid detection of pathogen nucleic acid. This paper introduces the biological mechanism and classification of CRISPR-Cas technology, summarizes the existing rapid detection technology for pathogen nucleic acid based on the trans cleavage activity of Cas, describes its characteristics, functions, and application scenarios, and prospects the future application of this technology.

## 1 Introduction

Based on the characteristics of Cas that specifically recognizes target nucleic acids or activates Incidental cutting activity after recognition, scientists have successfully developed a series of CRISPR-Cas technologies including *Specific High-sensitivity Enzymatic Reporter unlocking* (SHERLOCK), *DNA Endonuclease Targeted CRISPR Trans Reporter* (DETECTR) and *one-Hour Low-cost Multipurpose highly Efficient System* (HOLMES) (Abudayyeh et al., 2017; Stower, 2018; Kaminski et al., 2021). CRISPR-Cas technology has the characteristics of rapidity, accuracy, sensitivity, simplicity, and economy. It has been successfully applied to the detection of pathogenic microorganisms, genetic diseases, tumor gene mutations, small molecules, etc. It is an ideal next-generation rapid and sensitive nucleic acid on-site detection technology. Fast, convenient, and intelligent are the pain points of on-site nucleic acid detection. Creating intelligent nucleic acid detection products based on CRISPR-Cas are the future development direction of the field (Yang and Rothman, 2004). Rapid and sensitive diagnosis has a huge social demand, especially since the COVID-19 epidemic poses a huge threat to human society, which makes people more deeply realize the importance and necessity of establishing rapid and sensitive detection technology (Weissleder et al., 2020). Due to the rapid development of genomics, transcriptomics, and sequencing technology, nucleic acid diagnosis has become the leading role in the development of rapid and sensitive diagnosis. In recent years, gene editing, represented by CRISPR-Cas, has brought revolutionary progress in biotechnology (Pickar-Oliver and Gersbach, 2019). It is considered to be a revolutionary technology equivalent to *Polymerase Chain Reaction* (PCR) (Bodulev and Sakharov, 2020). Like PCR technology, it affects many aspects of life medicine and was rated as the first annual breakthrough scientific progress by *Science* twice in 2015 and 2017 (Pardee et al., 2016; Jackson et al., 2017). It won the first of Nature’s five most influential scientific events in the past 10 years and the Nobel Prize in Chemistry in 2020 (Makarova et al., 2020; Altae-Tran et al., 2021). At the same time, researchers have developed nucleic acid detection technology based on CRISPR-Cas, which is fast, accurate, sensitive, and economical, and is an ideal next-generation rapid and sensitive nucleic acid on-site detection technology. This technology was rated by *Science* as one of the top ten breakthroughs in science and technology in 2018 and *Nature* as one of the seven technologies worth paying attention to in 2022 (Coelho et al., 2022).

## 2 Biological mechanism of CRISPR-Cas system

**FIGURE 1:**
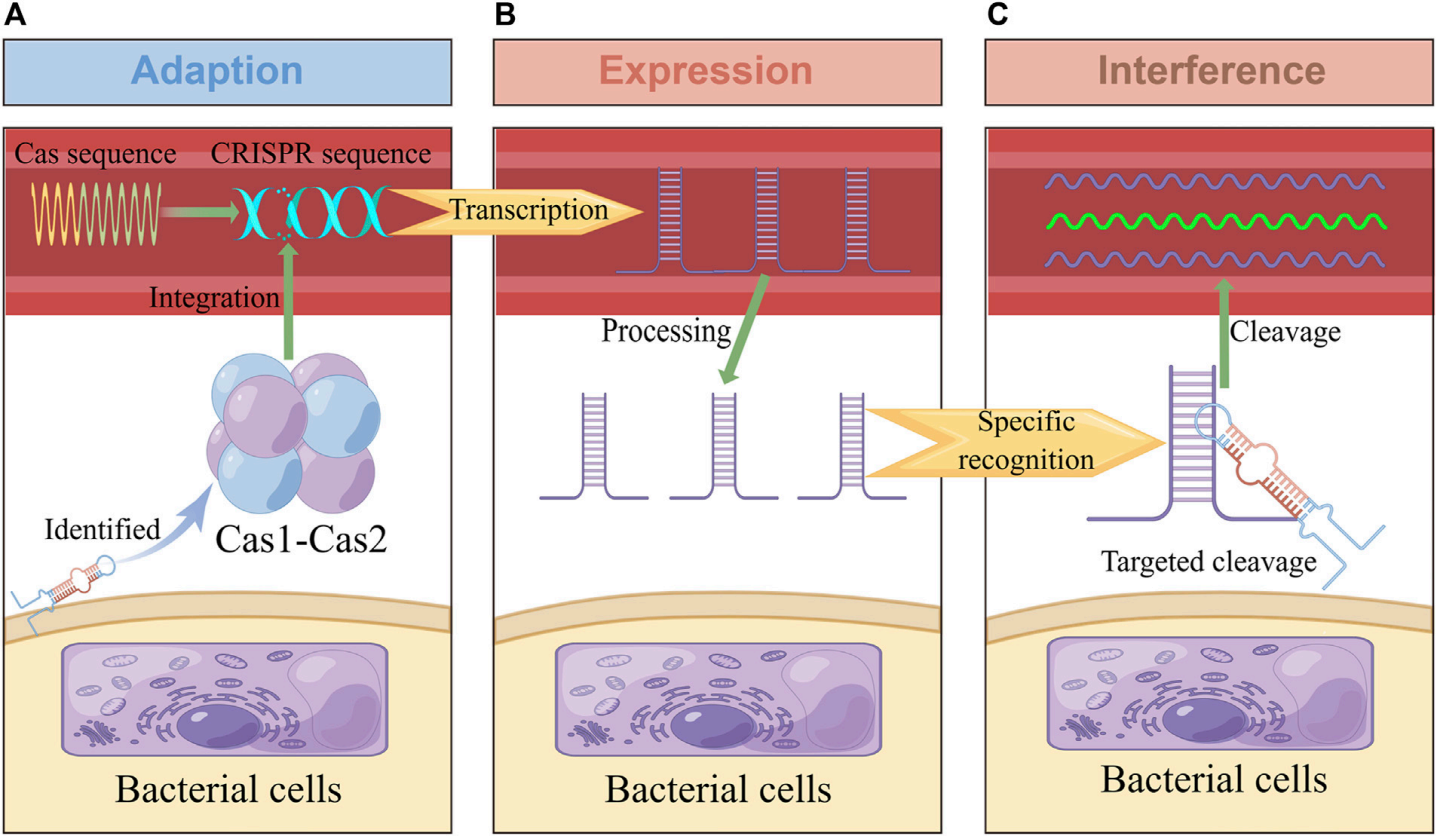
Adaptive immune response in the CRISPR-Cas system. This figure was drawn by Figdraw. **(A)** In the adaption stage, some short fragments of viruses, plasmids and other foreign nucleic acids are integrated into CRISPR repeats to form spacer sequences. **(B)** In the expression stage, CRISPR sequences were processed into crRNA containing spacer and repeat sequences, which were combined with CRISPR-associated effector proteins to form complexes. **(C)**: In the interference stage, the complex performs specific cleavage and inactivation of foreign nucleic acids complementary with crRNA sequences, so as to protect itself from viruses, plasmids and other invasion.

In 1987, the CRISPR site was found in the bacterial genome (Ishino et al., 1987), and Cas was found in 2002 (Jansen et al., 2002). It was later confirmed that the CRISPR-Cas system is an RNA-guided adaptive immune system that can resist viruses, plasmids, and other invasive genetic elements. Its immune process can be roughly divided into three stages: adaption, expression, and interference (Figure 1) (Jackson et al., 2017; Yao et al., 2018). Firstly, at the adaption stage, Cas recognizes and captures foreign nucleic acid fragments, acquires new spacer sequences, and integrates them into its own CRISPR array to form immune memory. Secondly, in the expression stage, when foreign nucleic acids invade again, the corresponding spacer sequence in the CRISPR array is transcribed to produce the precursor of *CRISPR RNA* (crRNA) and processed to obtain small, mature crRNA, which contains a conservative repeat sequence and a spacer sequence. The crRNA further interacts with one or more Cas used to form RNP (Ribonucleoprotein) complex (Hille et al., 2018). Thirdly, at the interference stage, Cas recognize the target nucleic acid through crRNA and are mediated to specifically destroy the invading nucleic acid (Jiao et al., 2021). In different CRISPR-Cas systems, Cas1 and Cas2 involved in the adaption stage are highly conserved to a large extent. In contrast, other Cas have significant differences, as they involve expression and interference stages (Makarova et al., 2015).

## 3 Classification of CRISPR-Cas system

**FIGURE 2:**
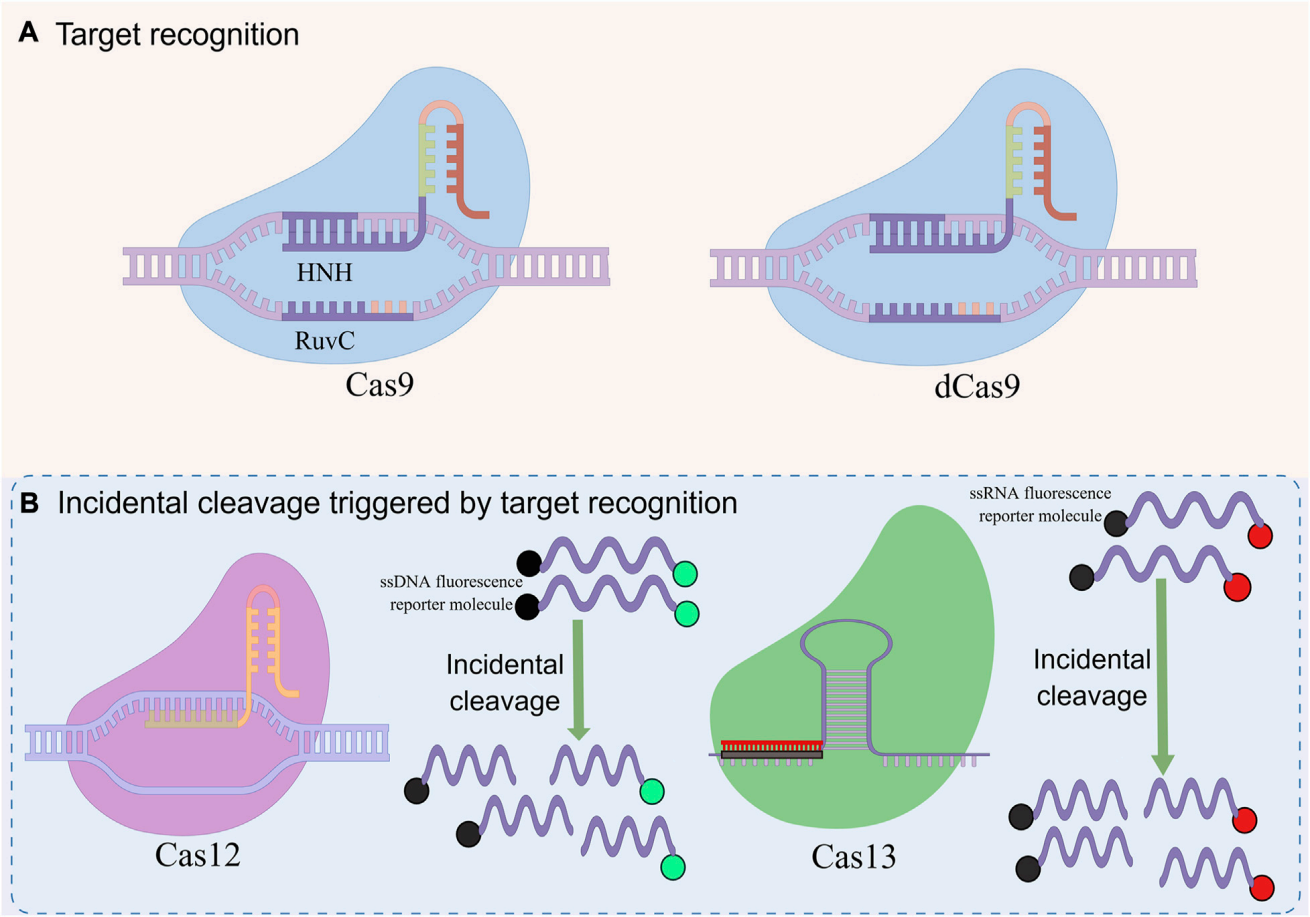
Basic principles of CRISPR-mediated nucleic acid detection. This figure was drawn by Figdraw. **(A)** RNA-guided target recognition system, which specifically recognizes and cleavages the target sequence containing PAM sites for detection by RNA-guided effector proteins. **(B)** Incidental cleavage system triggered by targeted recognition, after specifically recognizing and cleaving the target sequence, incidental cleavage of the surrounding labeled single-stranded nucleic acid is performed to generate detection signals for detection.

CRISPR-Cas system is divided into 2 categories, 6 types and 48 subtypes (Makarova et al., 2020). The first category can be divided into three types: type I, III, and IV, which use the complex composed of multiple Cas and crRNA to cut the target nucleic acid sequence (Makarova et al., 2017). The second category also includes three types: II, V, and VI, which have a single multifunctional Cas and crRNA for interference (Shmakov et al., 2015). This type of system has been widely used in the detection of pathogen nucleic acid, especially CRISPR-Cas9, Cas12, and Cas13 (Figure 2) (Li et al., 2021).

### 3.1 CRISPR-Cas system category I

There are three types of CRISPR-Cas system category I, which have effect complexes composed of *CRISPR-associated complex for antiviral defense* (Cascade), which is a complex composed of Cas and crRNA (Brouns et al., 2008). Cascade recognizes the *protospacer adjacent motif* (PAM) sequence, targets DNA target sites through crRNA, and uses Cas3 to achieve target site cutting (Hayes et al., 2016). At present, among the seven identified subtypes (I-A to I-F and I-U) (Zheng et al., 2020), subtype I-E CRISPR-Cas3 system also has trans cleavage activity (Yoshimi et al., 2022). The function of CRISPR-Cas3 is based on a multi-subunit complex composed of crRNA and CRISPR-Cas complex in the III-A subtype system, and the signature protein is Cas10. Type III overall composition and structure are highly similar to type I affect complex (Zhao et al., 2014). The target recognition of type III system can activate the polymerase activity of Cas10, and then Cas10 mediates the production of *cyclic oligoadenylate* (cOA), thus cutting target RNA and other adjacent RNA molecules (Kazlauskiene et al., 2017). Type IV CRISPR-Cas system is divided into IV-A, IV-B, and IV-D subtypes, which mainly exist in plasmids. The study found that type IV CRISPR-Cas system has strong targeting to plasmids. Some plasmids can use type IV CRISPR-Cas system to fight against the competition of other plasmids against the same host bacteria. Type IV CRISPR-Cas system may have the potential to be used in clinical drug-resistance gene therapy (Pinilla-Redondo et al., 2020).

### 3.2 CRISPR-Cas system category II

CRISPR-Cas system category II has unique effector proteins (Shmakov et al., 2017). Compared with CRISPR-Cas system category I, the effector module of CRISPR-Cas system category II is only a single protein with multiple domains and functions. Cas9 is a double RNA-guided DNA endonuclease, which mediates Cas9 to recognize the PAM sequence (5ʹ-NGG-3ʹ). Then, use the HNH and RuvC domains of Cas9 to cut the target double-stranded DNA, resulting in flat end double-stranded breaks (Shmakov et al., 2015). Recently, Jinek et al. (Jinek et al., 2012) constructed the *single-guide RNA* (sgRNA) to simplify CRISPR-Cas genome editing by reducing the three components system (Cas9, crRNA, trans-activating crRNA. Among them, trans-activating crRNA is abbreviated as tracrRNA) to two components (Cas9 and sgRNA) (Jiang et al., 2013). Type V CRISPR-Cas system can be divided into V-A, V-B, and V-C subtypes, and its effectors are Cas12a (Cpf1), Cas12b (C2c1), Cas12c (C2c3), etc. (Makarova et al., 2020). Type V CRISPR-Cas system only needs Cas and crRNA to edit the target site. After the crRNA recognizes the PAM site (5'- TTTN-3') and fully pairs with the target DNA base, Cas12a uses the RuvC domain to cut the target sequence in a cis manner to generate the 5 'sticky end, and at the same time, uses the trans cutting activity to cut any adjacent single-stranded DNA (ssDNA) (Swarts and Jinek, 2019). The feature makes the CRISPR-Cas12 system a hot spot in the field of nucleic acid detection. Recently, the system has also been applied to the detection of molecular markers, such as microRNA (Zeng et al., 2022), cardiac troponin I (cTn I) (Chen H. et al., 2022), etc. The research in this field is of great significance to achieve accurate *in vitro* diagnosis. In addition, Cas12 is often used Genetic Engineering (Kim et al., 2016; Xin et al., 2022). Like Cas12a, Cas14a belongs to the class 2 system type V family. Cas14a proteins have RNA-guided ssDNA-targeted endonuclease activity. Cas14a proteins are not required to recognize PAM sites in the DNA sequence (Aquino-Jarquin, 2019; Yuan et al., 2022). Type Ⅵ CRISPR-Cas system can be divided into Ⅵ-A, Ⅵ-B, Ⅵ-C, and Ⅵ-D subtypes. The signature protein is Cas13, which has *higher eukaryotes and prokaryotes nucleotide-binding* (HEPN) domains (Anantharaman et al., 2013). The uniqueness of this system is that Cas13 can recognize single-stranded RNA molecular targets. Under the targeting effect of crRNA, the Cas13-crRNA complex recognizes the sequence of the *Protospacer flanking site* (PFS) on the target nucleic acid, and at the same time cutting the target RNA, trans cuts the single-strand RNA (Abudayyeh et al., 2017; Cox et al., 2017). To sum up, the CRISPR-Cas system is diverse, and its key elements are different in composition, structure, and mechanism of action (Makarova et al., 2018). The in-depth exploration of the CRISPR-Cas system may provide a direction for the development of new diagnostic platforms.

## 4 Detection of pathogen nucleic acid based on trans cleavage activity

CRISPR-Cas was originally used as a gene editing system, which was widely studied and applied in the field of synthetic biology. According to the biological functions and characteristics of different proteins, a large number of new gene editing elements and tools were developed and designed (Cong et al., 2013). In 2016, the CRISPR-Cas system was first applied to nucleic acid detection and showed efficient and accurate pathogenic nucleic acid detection in subsequent research and development (Pardee et al., 2016). As a new interdisciplinary, synthetic biology focuses on the design of biomolecules or biological systems, which also provides new ideas and opportunities for molecular diagnosis (Hilton et al., 2015). The early nucleic acid detection technology mainly based on type II CRISPR-Cas9, but some technologies have no obvious advantages (Quan et al., 2019). With the continuous exploration of the CRISPR-Cas system, especially the discovery of the trans cleavage activity of Cas12, Cas13, and Cas14. With the application scope of the CRISPR-Cas system has been greatly expanded, and the field of nucleic acid detection has also been further innovated, which may open another door for research into the CRISPR-Cas system (Table 1). The characteristics of several nucleic acid detection methods based on the CRISPR-Cas system are briefly summarized from the aspects of amplification method, limit of detection, reaction time, detection of pathogens, signal reading method, and whether is it one-tube method? it is according to the classification of single CRISPR-associated effector proteins currently in common use (Table 2).

**TABLE 1:** Technologies for detecting pathogens based on the CRISPR-Cas system.

Main types of CRISPR	Technology item	Effector proteins	Target molecule	Mode of amplification	Limit of detection	Pathogens	Detection technologies
Type Ⅴ	DETECTR	Cas12a	DNA, RNA	RPA	αmol/L	HPV16/18 SARS-CoV-2	Fluorescence signal
	OR-DETECTR	Cas12a	RNA	RT-RPA	1-2.5 copies/μL	SARS-CoV-2 H1N1	Fluorescence signal
	HOLMES	Cas12a	DNA, RNA	PCR	αmol/L	JEV	Fluorescence signal
	HOLMES v2	Cas12b	DNA, RNA	LAMP	αmol/L	JEV	Fluorescence signal
	E-CRISPR	Cas12a	DNA	—	pmol/L	HPV1, B19	Electrochemistry
	CRISPR-ENHAN	LbCas12a	RNA	RT-LAMP	—	SARS-CoV-2, HIV, HCV	Lateral flow immunoassay
	AIOD-CRISPR	LbCas12a	RNA	RPA	1-2.5 copies/μL	SARS-CoV-2	Fluorescence signal
	SCAN	Cas12a	DNA, RNA	RT-PCR/RT-RPA	13.5 copies/μL	HIV	Nanopore sensor
	TB-QUICK	Cas12b	DNA	LAMP	1.3 copies/μL	*Mycobacterium tuberculosis*	Fluorescence signal
	DETECTR-Cas14	Cas14a	DNA, RNA	RPA	αmol/L	Viruses, Bacteria	Fluorescence signal
Type VI	SHERLOCK	Cas13a	DNA, RNA	RPA	αmol/L	Viruses, Bacteria	Fluorescence signal
	HUDSON	Cas13a	RNA	RT-RPA	1 copies/μL	Zika virus, Dengue virus	Fluorescence signal
	OR-SHERLOCK	Cas13a	RNA	RT-RPA	1–2 copies/μL	SARS-CoV-2	Fluorescence signal
	SHERLOCK v2	Cas12a	DNA, RNA	RPA	—	SARS-CoV-2	Lateral flow immunoassay

**TABLE 2:** Characteristics of nucleic acid detection methods based on CRISPR-Cas system.

Effector proteins	Amplification method	Limit of detection	Pathogens	Time (min)	Read signal	One-tube
Cas9	NASBA	1 fmol	Zika virus	180	Paper sensors	No
dCas9	PCR	50 fmol	*Mycobacterium tuberculosis*	50–60	Fluorescence	No
	RPA	1 αmol	HPV	60	Fluorescence	No
Cas12a	LAMP	1 copy/µL	HBV	13–20	Fluorescence, Lateral flow immunoassay	No
	RPA	50 CFU/mL	*Mycobacterium tuberculosis*	90	Fluorescence	No
	RPA	10 copies/µL	SARS-CoV-2	40	Fluorescence, Lateral flow immunoassay	No
	PCR	1 αmol	JEV	60	Fluorescence	No
Cas12b	LAMP	1 αmol	JEV	60	Fluorescence	Yes
Cas13a	RPA,	1 αmol	Zika virus, Dengue virus	30–60	Lateral flow immunoassay	No
	HCR	1 αmol	SARS-CoV-2	60	Fluorescence	No
Cas14a	RPA	1 αmol	E.coli O157:H7	120	Fluorescence	No

### 4.1 CRISPR-Cas9

Cas9 is a marker protein in the type II CRISPR-Cas system, which is the most widely studied and applied protein at present. The CRISPR-Cas9 system requires a *single-guide RNA* (sgRNA) chimeric with crRNA and tracrRNA to guide Cas9 to specifically recognize and cut targeted double-stranded DNA containing PAM sites (Sternberg et al., 2014; Najah et al., 2019). According to different gene fragments, complementary sgRNAs can be designed to specifically recognize and cut different target sequences. Pardee et al. (Pardee et al., 2016) took the lead in combining the CRISPR-Cas system with *Nucleic Acid Sequence-based Amplification* (NASBA) technology for Zika virus detection in 2016, with a detection limit of 1 fmol and the ability to detect and distinguish different virus subtypes with single base differences, as well as dengue virus infection samples with clinical symptoms similar to Zika virus. Its advantage is that the detection system is freeze-dried on the test paper, which is economical, portable, and can be stored for a long time. It is suitable for on-site and resource-scarce areas. However, the long detection time (180 min) is the main reason that hinders its large-scale application. Then researchers tried to combine the CRISPR-Cas9 system with an isothermal exponential amplification reaction, which improved amplification efficiency and shortened detection time while maintaining high sensitivity and specificity. Huang et al. (2018) combined the CRISPR-Cas9 system with an isothermal exponential amplification reaction to detect DNA methylation and total RNA of *Listeria* monocytogenes. The detection limit of this method is 0.82 αmol, and it shows high specificity in distinguishing single base mismatch. Subsequently, Wang et al. (Ting et al., 2019) developed isothermal exponential amplification reaction based on the CRISPR-Cas9 system, which can achieve specific detection of typhoid *bacillus* within 60 min, and the results can be detected if there are two copies in the 20 µL reaction system; Its disadvantage is that it relies on expensive real-time fluorescence reading equipment to read results, which is not suitable for detection in resource-poor areas. Quan et al. (2019) combined the CRISPR-Cas9 system with PCR technology to develop the method of finding low abundance sequences through hybridization, which is used to detect pathogens resistant to antimicrobial therapy and has been successfully applied to the falciparum malaria model, providing the possibility for CRISPR-Cas technology to be applied to other parasite detection. *Nuclease deficient Cas9* (dCas9) is chemically modified Cas9, which is characterized by loss of endonuclease activity but still can specifically recognize and bind target sequences. Zhang et al. (2022) used the paired CRISPR-dCas9 system and PCR technology for the detection of *mycobacterium tuberculosis* and labeled the paired dCas9 with luciferase as a guide. When RNA guides the dCas9 pair to specifically bind to the same target, and the distance between the two binding sites is 19–23 bases. The fluorescein is oxidized, emitting fluorescence signals and detecting the pathogen. In addition to using fluorescent labeling, Hajian et al. (2019) combined the CRISPR-Cas system with the electronic transistor made of graphene and developed a biosensor: that is, the dCas9 is fixed on the graphene transistor. After adding DNA samples, dCas9 combines with the target DNA to change the conductivity of graphene and the electrical characteristics of the transistor, to achieve rapid sample detection. The sensitivity of this method is as low as 1.7 fmol, and the detection time is only 15 min. These studies demonstrated the potential of dCas9 as a powerful platform for *in vitro* nucleic acid detection, but its detection sensitivity still needs to be improved.

### 4.2 CRISPR-Cas12

Cas12 belongs to type V CRISPR-Cas system. At present, there are much researches on Cas12a (also known as Cpf1) and Cas12b. Unlike Cas9, which requires two nuclease domains (HNH and RuvC) to exert cutting activity and produce flat-end incisions, Cas12a requires only one nuclease domain (RuvC) to produce sticky-end incisions; Its target is double-stranded DNA and single-stranded DNA (Zetsche et al., 2015). In 2018, researchers found that after Cas12a specifically cut target DNA under the guidance of crRNA, it also cut nontargeted single-strand DNA nearby (Li et al., 2018a). Based on this “accessory cutting” feature, Chen et al. (2018) combined *recombinase polymerase amplification* (RPA) with the CRISPR-Cas12a system to develop DETECTR nucleic acid detection technology successfully detects *human papillomavirus* (HPV) type 16 and 18 and realizes the differentiation of different subtypes. In this method, RPA is used to pre-amplify the target DNA, and then the CRISPR-Cas12a system is added. After the crRNA in this system guides Cas12a to specifically recognize and cut the target DNA, nonspecifically cut the surrounding fluorescent group labeled single-strand DNA and generate fluorescence signals. DETECTR has extremely high sensitivity and specificity, its detection limit has reached αmol level, and the detection results have also been confirmed to be in good agreement with the PCR detection results. The detection method based on the DETECTR system can achieve high sensitivity and specificity for norovirus, HPV, metapneumovirus, and other viruses (Qian et al., 2021a; Qian et al., 2021b). Ding et al. (2021) combined *Reverse Transcription Loop-mediated Isothermal Amplification* (RT-LAMP) with Cas12a to achieve rapid detection of HBV in 13–20 min, with a detection limit of 1 copy/µL. Based on the DETECTR detection platform, a research team combined CRISPR-Cas12a technology with RPA to achieve rapid and accurate detection of food-borne bacteria, including *Escherichia coli*, *Listeria* monocytogenes, *Staphylococcus aureus*, and *Vibrio* parahaemolyticus, as well as *Mycobacterium tuberculosis* and *Mycoplasma* pneumonia, with high sensitivity and specificity (Ai et al., 2019; Bhattacharjee et al., 2022; Li et al., 2022).In addition, the DETECTR system is also widely used for the detection of SARS-CoV-2. After reverse transcription and amplification of purified RNA from the nasopharynx or oropharynx swabs by reverse transcription isothermal amplification technology, the CRISPR-Cas system specifically recognizes and cuts the target to generate fluorescence signals, thereby confirming the existence of SARS-CoV-2. For example, Broughton et al. (2020) combined the CRISPR-Cas12a system with RT-LAMP to develop a DETECTR detection platform for SARS-CoV-2. However, the DETECTR method is divided into two steps, which are prone to cross-contamination. To avoid this possibility, Li et al. (Li et al., 2018b) used physical methods to put the isothermal amplification system and CRISPR-Cas12a system in the same test tube and separate them. After 15 min of isothermal amplification reaction, the two systems were mixed, thus realizing one-step detection and simplifying the operation steps while avoiding cross-contamination. In recent years, a research team has established a light-activated, one-step RPA-CRISPR-Cas method that was successfully used for the rapid detection of the African swine fever virus. The detection time was only 40 min, and the detection sensitivity was up to 2.5 copies (Chen Y. et al., 2022). Compared with the conventional multi-step detection method that first performs isothermal amplification and then adds CRISPR-Cas system, this detection method greatly simplifies the operation process on the premise of consistent sensitivity. Ding et al. (2020) developed *An Integrated Dual CRISPR-Cas12a* (AIOD CRISPR, all in one dual CRISPR-Cas12a) system, integrating the amplification system and CRISPR-Cas system into the same reaction system so that there is no need to amplify and transfer amplification products separately; At the same time, the system uses double crRNA to detect SARS-CoV-2 and HPV1, which is more simple, more sensitive and more specific. Cas12b has the same cutting activity as Cas12a, but its optimal reaction temperature is relatively higher, its specificity is higher, and its requirements for reaction conditions are more stringent. Li et al. (2019) combined PCR with the CRISPR-Cas12a system and developed HOLMES, but the system also requires a two-step reaction. To solve this problem, the research team developed HOLMES v2, which integrates the RT-LAMP system and the CRISPR-Cas12b system with relatively higher optimal reaction temperature into one reaction system, realizing one-step detection (Wang et al., 2019). The HOLMES series of methods established by the team successfully achieved αmol level detection of Japanese encephalitis virus because the reaction conditions of Cas12b are stricter, so the research and application of Cas12b are less than that of Cas12a.

### 4.3 CRISPR-Cas13

Cas13 belongs to type VI CRISPR-Cas system, which has RNA nuclease activity and only works on single-stranded RNA. Cas13a, also known as C2c2, is the first effector protein used to target RNA cleavage (Cox et al., 2017; O'Connell, 2019). Cas13a also has the activity of “incidental cleavage”. Under the guidance of crRNA, Cas13a specifically cleaves targeted single-strand RNA by relying on the flanking sequence (PFS sequence, which has the same function as the PAM sequence) of the anterior spacer sequence, and then performs non-specific cleavage on the nearby single-strand RNA (Abudayyeh et al., 2016). Abudayyeh et al. (2019) combined CRISPR-Cas13a technology with RPA to develop a SHERLOCK nucleic acid detection system, which can detect Zika virus as low as 2 αmol; Its specificity is also very high, and it can recognize single nucleotide mismatch. Then Myhrvold et al. (2018) developed a SHERLOCK v2 nucleic acid detection system based on a strip by using a biotin-labeled reporter gene, which realized simple and rapid detection of the Zika virus, and its detection results were consistent with RT-PCR detection results. Compared with the detection method based on PCR technology, the detection platform based on SHERLOCK and SHERLOCK v2 does not need thermal cycles and complex laboratory systems, and it can be combined with technologies such as test strips, which makes reading results easier and faster, and has higher sensitivity and specificity. Studies have shown that DETECTR and SHERLOCK diagnostic kits for SARS-CoV-2 detection have been approved and marketed abroad (Guo et al., 2020; Hou et al., 2020; Pang et al., 2020). In addition, the detection platform based on SHERLOCK, other researchers combined CRISPR-Cas13a with the general autonomous enzyme-free *Hybridization Chain Reaction* (HCR) and triggered the downstream HCR circuit by designing the release promoter sequence of the lysate probe report, to detect SARS-CoV-2 or related coronavirus strains, with a sensitivity of αmol. The reaction time is shorter than 60 min (Yang et al., 2021; Li et al., 2023).

### 4.4 CRISPR-Cas14

Cas14 protein, also referred to as Cas12f, is the shortest member of the CRISPR-associated protein family, consisting of approximately 400–700 amino acids (Zhang and An, 2022). There are three subtypes of Cas14: Cas14a, Cas14b, and Cas14c. It has the ability to cleave specific nucleic acid sequences (cis-cleavage) and non-specific single-stranded DNA and RNA sequences (trans-cleavage). When a small guide RNA (sgRNA) is present, these subtypes are able to cleave ssDNA without the need for a PAM sequence (crRNA) (Ma et al., 2020). It is estimated that more than CRISPR-Cas14 system has evolved independently, although the Cas14 proteins display a significant amount of sequence diversity. There exhibit a common RuvC nuclease domain, which is universally found in V-type CRISPR-Cas system (Harrington et al., 2018). In addition to enabling the advancement of nucleic acid targeted editing, virus detection, and nucleic acid detection through protein purification, it facilitates the development of nucleic acid targeted editing techniques (Harrington et al., 2018; Aquino-Jarquin, 2019; He et al., 2023; Li et al., 2023).

## 5 Discussion

CRISPR-Cas system, as a novel and powerful nucleic acid diagnostic tool, has developed many detection technologies based on the inherent characteristics of different Cas, and the detection technology based on Cas12 and Cas13 has the most potential. DETECTR–Cas14 technology combines RPA and Cas14 technology, has greater specificity than the previous DETECTR–Cas12 technology, which has been applied successfully to the typing of SNP genes (Harrington et al., 2018). So far, DETECTR (Chen et al., 2018), SHERLOCK (Gootenberg et al., 2018), and many other detection technologies have been applied to the detection of a variety of pathogens, including SARS-CoV-2 (Kellner et al., 2019; Khan et al., 2021). These detection methods can usually reach the sensitivity of fmol/L to αmol/L and achieve a single base resolution. To simplify and optimize the detection process, researchers have developed some simple sample pretreatment, such as *Heating Unextracted Diagnostic Samples to Obliterate Nucleases* (HUDSON) (Barnes et al., 2020), which can directly amplify and detect pathogenic nucleic acids (Gootenberg et al., 2018). The development of one-tube reaction technology has reduced the risk of sample contamination, and the target nucleic acid amplification and Cas cutting are conducted in a closed tube; The development of different result reading methods (fluorescent reading, colorimetric reading, electrochemical reading, etc.), and the combination of portable low-cost instruments (smartphones, etc.), which are very suitable for on-site detection. In addition, in some detection technologies, the design and use of optimized crRNA reduced the miss effect, and the use of Cas with lower nucleotide mismatch tolerance also solved the potential false positive problem. Although the CRISPR-Cas system has many advantages, there are still many challenges to overcome before the technology is translated into clinical application. For example, the recognition and cleavage of target nucleic acid by Cas12, Cas13, Cas14, and other proteins depends on PAM or PFS sequence, which limits the selection of target nucleic acid region and greatly reduces the selection range of primer design. Moreover, the existing detection technologies cannot get rid of the nucleic acid pre-amplification step and realize one-tube multiple detections. At the same time, the detection technology developed based on CRISPR-Cas system, like PCR technology, cannot monitor the possible emerging virus threat or pandemic in the future (Yang and Rothman, 2004). While there are so much restriction, but there is still a strong need to develop large-scale multiplexed nucleic acid detection technology for the CRISPR-Cas system (Ackerman et al., 2020). Because the CRISPR-Cas technology is a new method for molecular biology detection that offers many advantages over traditional PCR methods. For example, it does not require any expensive equipment and consumables, is easier to operate and saves time than traditional PCR technology (Li et al., 2023).

## 6 Conclusion and prospects

Nucleic acid detection based on the CRISPR system is still in its infancy, and it still faces the following challenges to promote to the field and clinical application: Firstly, the carrier function of the CRISPR-Cas system is limited by the size of pathogen genes, and most of the currently developed Cas are large molecular weight proteins; Secondly, Type Ⅱ CRISPR-Cas requires a PAM or PFS site in the target binding site to activate the cutting activity, which limits CRISPR-Cas system to be used for short target sequence detection; Thirdly, at present, most of them are based on the detection technology of CRISPR-Cas system requires pre-amplification of nucleic acid before detection, which adds operation steps. Based on the above challenges, the following work can be carried out in the future: Firstly, try to develop and utilize new Cas effector proteins with smaller molecular weight, such as Cas14 (Harrington et al., 2018; Khan et al., 2019; Yang et al., 2021)CAS; Secondly, through primer design, the PAM sequence was artificially inserted to broaden the target detection range; Thirdly, at present, a series of one-step CRISPR-Cas detection platforms have been developed to simplify the operation steps. We can also try to build efficient microfluidic enrichment and other technologies, improve the digestion activity of Cas effector proteins, improve the target capture efficiency, and achieve rapid and sensitive detection. Fourthly, develop CRISPR-Cas system in combination with more other nucleic acid amplification methods to improve the detection ability of very low concentration nucleic acids; Fifthly, the one-tube equal temperature detection technology has promoted the development of fast and convenient detection methods, making it possible to visually detect viral RNA, including test strips, portable UV lamps, color changes, etc. ; Sixthly, develop one-tube thermostatic non fluorescent detection methods combined with isothermal amplification and CRISPR-Cas, such as electrochemical (Heo et al., 2022) or colorimetric (Zhang et al., 2021) detection methods, to improve the matrix tolerance of nonfluorescent detection systems; Seventhly, develop new storage and use methods, such as paper based lyophilized reagent (Pardee et al., 2016), to avoid reducing the activity of reaction reagent during storage, transportation and use; Eighthly, develop more convenient and efficient sample pretreatment methods, reduce operation process and time, and apply to different types of samples; Ninthly, develop high-throughput detection methods, develop detection probes with multiple fluorescent channels or combine them with high-throughput detection platforms, such as micro-drop digital CRISPR with the optimization and further understanding of CRISPR-Cas system, detection technology developed based on CRISPR-Cas system may become one of the mainstream platforms for pathogen nucleic acid detection. Although many detection technologies cannot be quickly transferred from laboratory to clinical application and play their roles shortly, but they can provide a good platform for large-scale population screening, better and faster control of pathogen transmission, and rapid on-site detection (Patchsung et al., 2020).

## References

[B1] Abudayyeh O. O. Gootenberg J. S. Essletzbichler P. Han S. Joung J. Belanto J. J. (2017). RNA targeting with CRISPR-Cas13. Nature 550 (7675), 280–284. 10.1038/nature24049 28976959 PMC5706658

[B2] Abudayyeh O. O. Gootenberg J. S. Franklin B. Koob J. Kellner M. J. Ladha A. (2019). A cytosine deaminase for programmable single-base RNA editing. Sci. (New York, NY) 365 (6451), 382–386. 10.1126/science.aax7063 PMC6956565 31296651

[B3] Abudayyeh O. O. Gootenberg J. S. Konermann S. Joung J. Slaymaker I. M. Cox D. B. (2016). C2c2 is a single-component programmable RNA-guided RNA-targeting CRISPR effector. Sci. (New York, NY) 353 (6299), aaf5573. 10.1126/science.aaf5573 PMC5127784 27256883

[B4] Ackerman C. M. Myhrvold C. Thakku S. G. Freije C. A. Metsky H. C. Yang D. K. (2020). Massively multiplexed nucleic acid detection with Cas13. Nature 582 (7811), 277–282. 10.1038/s41586-020-2279-8 32349121 PMC7332423

[B5] Ai J. W. Zhou X. Xu T. Yang M. Chen Y. He G. Q. (2019). CRISPR-based rapid and ultra-sensitive diagnostic test for *Mycobacterium tuberculosis* . Emerg. Microbes Infect. 8 (1), 1361–1369. 10.1080/22221751.2019.1664939 31522608 PMC6758691

[B6] Altae-Tran H. Kannan S. Demircioglu F. E. Oshiro R. Nety S. P. McKay L. J. (2021). The widespread IS200/IS605 transposon family encodes diverse programmable RNA-guided endonucleases. Sci. (New York, NY) 374 (6563), 57–65. 10.1126/science.abj6856 PMC8929163 34591643

[B7] Anantharaman V. Makarova K. S. Burroughs A. M. Koonin E. V. Aravind L. (2013). Comprehensive analysis of the HEPN superfamily: identification of novel roles in intra-genomic conflicts, defense, pathogenesis and RNA processing. Biol. Direct 8, 15. 10.1186/1745-6150-8-15 23768067 PMC3710099

[B8] Aquino-Jarquin G. (2019). CRISPR-Cas14 is now part of the artillery for gene editing and molecular diagnostic. Nanomedicine 18, 428–431. 10.1016/j.nano.2019.03.006 30935995

[B9] Barnes K. G. Lachenauer A. E. Nitido A. Siddiqui S. Gross R. Beitzel B. (2020). Deployable CRISPR-Cas13a diagnostic tools to detect and report Ebola and Lassa virus cases in real-time. Nat. Commun. 11 (1), 4131. 10.1038/s41467-020-17994-9 32807807 PMC7431545

[B10] Bhattacharjee R. Nandi A. Mitra P. Saha K. Patel P. Jha E. (2022). Theragnostic application of nanoparticle and CRISPR against food-borne multi-drug resistant pathogens. Mater Today Bio 15, 100291. 10.1016/j.mtbio.2022.100291 PMC9194658 35711292

[B11] Bodulev O. L. Sakharov I. Y. (2020). Isothermal nucleic acid amplification techniques and their use in bioanalysis. Biochem. (Mosc) 85 (2), 147–166. 10.1134/S0006297920020030 PMC7223333 32093592

[B12] Broughton J. P. Deng X. Yu G. Fasching C. L. Servellita V. Singh J. (2020). CRISPR-Cas12-based detection of SARS-CoV-2. Nat. Biotechnol. 38 (7), 870–874. 10.1038/s41587-020-0513-4 32300245 PMC9107629

[B13] Brouns S. J. Jore M. M. Lundgren M. Westra E. R. Slijkhuis R. J. Snijders A. P. (2008). Small CRISPR RNAs guide antiviral defense in prokaryotes. Sci. (New York, NY) 321 (5891), 960–964. 10.1126/science.1159689 PMC5898235 18703739

[B14] Chen H. Li Z. Y. Chen J. Yu H. Zhou W. Shen F. (2022a). CRISPR/Cas12a-based electrochemical biosensor for highly sensitive detection of cTnI. Bioelectrochemistry 146, 108167. 10.1016/j.bioelechem.2022.108167 35623274

[B15] Chen J. S. Ma E. Harrington L. B. Da Costa M. Tian X. Palefsky J. M. (2018). CRISPR-Cas12a target binding unleashes indiscriminate single-stranded DNase activity. Sci. (New York, NY) 360 (6387), 436–439. 10.1126/science.aar6245 PMC6628903 29449511

[B16] Chen Y. Xu X. Wang J. Zhang Y. Zeng W. Liu Y. (2022b). Photoactivatable CRISPR/Cas12a strategy for one-pot DETECTR molecular diagnosis. Anal. Chem. 94 (27), 9724–9731. 10.1021/acs.analchem.2c01193 35762828

[B17] Coelho R. Tozzi A. Disler M. Lombardo F. Fedier A. López M. N. (2022). Overlapping gene dependencies for PARP inhibitors and carboplatin response identified by functional CRISPR-Cas9 screening in ovarian cancer. Cell Death Dis. 13 (10), 909. 10.1038/s41419-022-05347-x 36307400 PMC9616819

[B18] Cong L. Ran F. A. Cox D. Lin S. Barretto R. Habib N. (2013). Multiplex genome engineering using CRISPR/Cas systems. Sci. (New York, NY) 339 (6121), 819–823. 10.1126/science.1231143 PMC3795411 23287718

[B19] Cox D. B. T. Gootenberg J. S. Abudayyeh O. O. Franklin B. Kellner M. J. Joung J. (2017). RNA editing with CRISPR-Cas13. Sci. (New York, NY) 358 (6366), 1019–1027. 10.1126/science.aaq0180 PMC5793859 29070703

[B20] Ding R. Long J. Yuan M. Zheng X. Shen Y. Jin Y. (2021). CRISPR/Cas12-Based ultra-sensitive and specific point-of-care detection of HBV. Int. J. Mol. Sci. 22 (9), 4842. 10.3390/ijms22094842 34063629 PMC8125043

[B21] Ding X. Yin K. Li Z. Lalla R. V. Ballesteros E. Sfeir M. M. (2020). Ultrasensitive and visual detection of SARS-CoV-2 using all-in-one dual CRISPR-Cas12a assay. Nat. Commun. 11 (1), 4711. 10.1038/s41467-020-18575-6 32948757 PMC7501862

[B22] Gootenberg J. S. Abudayyeh O. O. Kellner M. J. Joung J. Collins J. J. Zhang F. (2018). Multiplexed and portable nucleic acid detection platform with Cas13, Cas12a, and Csm6. Sci. (New York, NY) 360 (6387), 439–444. 10.1126/science.aaq0179 PMC5961727 29449508

[B23] Guo L. Sun X. Wang X. Liang C. Jiang H. Gao Q. (2020). SARS-CoV-2 detection with CRISPR diagnostics. Cell Discov. 6, 34. 10.1038/s41421-020-0174-y 32435508 PMC7235268

[B24] Hajian R. Balderston S. Tran T. deBoer T. Etienne J. Sandhu M. (2019). Detection of unamplified target genes via CRISPR-Cas9 immobilized on a graphene field-effect transistor. Nat. Biomed. Eng. 3 (6), 427–437. 10.1038/s41551-019-0371-x 31097816 PMC6556128

[B25] Harrington L. B. Burstein D. Chen J. S. Paez-Espino D. Ma E. Witte I. P. (2018). Programmed DNA destruction by miniature CRISPR-Cas14 enzymes. Sci. (New York, NY) 362 (6416), 839–842. 10.1126/science.aav4294 PMC6659742 30337455

[B26] Hayes R. P. Xiao Y. Ding F. van Erp P. B. Rajashankar K. Bailey S. (2016). Structural basis for promiscuous PAM recognition in type I-E Cascade from *E. coli* . Nature 530 (7591), 499–503. 10.1038/nature16995 26863189 PMC5134256

[B27] He Y. Yan W. Long L. Dong L. Ma Y. Li C. (2023). The CRISPR/cas system: A customizable toolbox for molecular detection. Genes (Basel). 14 (4), 850. 10.3390/genes14040850 37107608 PMC10137550

[B28] Heo W. Lee K. Park S. Hyun K. A. Jung H. I. (2022). Electrochemical biosensor for nucleic acid amplification-free and sensitive detection of severe acute respiratory syndrome coronavirus 2 (SARS-CoV-2) RNA via CRISPR/Cas13a trans-cleavage reaction. Biosens. Bioelectron. 201, 113960. 10.1016/j.bios.2021.113960 35016109 PMC8730380

[B29] Hille F. Richter H. Wong S. P. Bratovič M. Ressel S. Charpentier E. (2018). The biology of CRISPR-cas: backward and forward. Cell 172 (6), 1239–1259. 10.1016/j.cell.2017.11.032 29522745

[B30] Hilton I. B. D'Ippolito A. M. Vockley C. M. Thakore P. I. Crawford G. E. Reddy T. E. (2015). Epigenome editing by a CRISPR-Cas9-based acetyltransferase activates genes from promoters and enhancers. Nat. Biotechnol. 33 (5), 510–517. 10.1038/nbt.3199 25849900 PMC4430400

[B31] Hou T. Zeng W. Yang M. Chen W. Ren L. Ai J. (2020). Development and evaluation of a rapid CRISPR-based diagnostic for COVID-19. PLoS Pathog. 16 (8), e1008705. 10.1371/journal.ppat.1008705 32853291 PMC7451577

[B32] Huang M. Zhou X. Wang H. Xing D. (2018). Clustered regularly interspaced short palindromic repeats/cas9 triggered isothermal amplification for site-specific nucleic acid detection. Anal. Chem. 90 (3), 2193–2200. 10.1021/acs.analchem.7b04542 29260561

[B33] Ishino Y. Shinagawa H. Makino K. Amemura M. Nakata A. (1987). Nucleotide sequence of the iap gene, responsible for alkaline phosphatase isozyme conversion in *Escherichia coli*, and identification of the gene product. J. Bacteriol. 169 (12), 5429–5433. 10.1128/jb.169.12.5429-5433.1987 3316184 PMC213968

[B34] Jackson S. A. McKenzie R. E. Fagerlund R. D. Kieper S. N. Fineran P. C. Brouns S. J. (2017). CRISPR-cas: adapting to change. Science 356 (6333), eaal5056. 10.1126/science.aal5056 28385959

[B35] Jansen R. Embden J. D. Gaastra W. Schouls L. M. (2002). Identification of genes that are associated with DNA repeats in prokaryotes. Mol. Microbiol. 43 (6), 1565–1575. 10.1046/j.1365-2958.2002.02839.x 11952905

[B36] Jiang W. Bikard D. Cox D. Zhang F. Marraffini L. A. (2013). RNA-guided editing of bacterial genomes using CRISPR-Cas systems. Nat. Biotechnol. 31 (3), 233–239. 10.1038/nbt.2508 23360965 PMC3748948

[B37] Jiao C. Sharma S. Dugar G. Peeck N. L. Bischler T. Wimmer F. (2021). Noncanonical crRNAs derived from host transcripts enable multiplexable RNA detection by Cas9. Sci. (New York, NY) 372 (6545), 941–948. 10.1126/science.abe7106 PMC8224270 33906967

[B38] Jinek M. Chylinski K. Fonfara I. Hauer M. Doudna J. A. Charpentier E. (2012). A programmable dual-RNA-guided DNA endonuclease in adaptive bacterial immunity. Sci. (New York, NY) 337 (6096), 816–821. 10.1126/science.1225829 PMC6286148 22745249

[B39] Kaminski M. M. Abudayyeh O. O. Gootenberg J. S. Zhang F. Collins J. J. (2021). CRISPR-based diagnostics. Nat. Biomed. Eng. 5 (7), 643–656. 10.1038/s41551-021-00760-7 34272525

[B40] Kazlauskiene M. Kostiuk G. Venclovas Č. Tamulaitis G. Siksnys V. (2017). A cyclic oligonucleotide signaling pathway in type III CRISPR-Cas systems. Sci. (New York, NY) 357 (6351), 605–609. 10.1126/science.aao0100 28663439

[B41] Kellner M. J. Koob J. G. Gootenberg J. S. Abudayyeh O. O. Zhang F. (2019). Sherlock: nucleic acid detection with CRISPR nucleases. Nat. Protoc. 14 (10), 2986–3012. 10.1038/s41596-019-0210-2 31548639 PMC6956564

[B42] Khan M. Z. Haider S. Mansoor S. Amin I. (2019). Targeting plant ssDNA viruses with engineered miniature CRISPR-cas14a. Trends Biotechnol. 37 (8), 800–804. 10.1016/j.tibtech.2019.03.015 31023561

[B43] Khan W. A. Barney R. E. Tsongalis G. J. (2021). CRISPR-cas13 enzymology rapidly detects SARS-CoV-2 fragments in a clinical setting. J. Clin. Virol. 145, 105019. 10.1016/j.jcv.2021.105019 34753073 PMC8553369

[B44] Kim D. Kim J. Hur J. K. Been K. W. Yoon S. H. Kim J. S. (2016). Erratum: genome-wide analysis reveals specificities of Cpf1 endonucleases in human cells. Nat. Biotechnol. 34 (8), 888. 10.1038/nbt0816-888a 27504781

[B45] Li F. Xiao J. Yang H. Yao Y. Li J. Zheng H. (2022). Development of a rapid and efficient RPA-CRISPR/Cas12a assay for Mycoplasma pneumoniae detection. Front. Microbiol. 13, 858806. 10.3389/fmicb.2022.858806 35369478 PMC8965353

[B46] Li L. Li S. Wu N. Wu J. Wang G. Zhao G. (2019). HOLMESv2: A CRISPR-cas12b-assisted platform for nucleic acid detection and DNA methylation quantitation. ACS Synth. Biol. 8 (10), 2228–2237. 10.1021/acssynbio.9b00209 31532637

[B47] Li P. Wang L. Yang J. Di L. J. Li J. (2021). Applications of the CRISPR-Cas system for infectious disease diagnostics. Expert Rev. Mol. Diagn 21 (7), 723–732. 10.1080/14737159.2021.1922080 33899643

[B48] Li S. Y. Cheng Q. X. Liu J. K. Nie X. Q. Zhao G. P. Wang J. (2018a). CRISPR-Cas12a has both cis- and trans-cleavage activities on single-stranded DNA. Cell Res. 28 (4), 491–493. 10.1038/s41422-018-0022-x 29531313 PMC5939048

[B49] Li S. Y. Cheng Q. X. Wang J. M. Li X. Y. Zhang Z. L. Gao S. (2018b). CRISPR-Cas12a-assisted nucleic acid detection. Cell Discov. 4, 20. 10.1038/s41421-018-0028-z 29707234 PMC5913299

[B50] Li X. Zhu S. Zhang X. Ren Y. He J. Zhou J. (2023). Advances in the application of recombinase-aided amplification combined with CRISPR-Cas technology in quick detection of pathogenic microbes. Front. Bioeng. Biotechnol. 11, 1215466. 10.3389/fbioe.2023.1215466 37720320 PMC10502170

[B51] Ma P. Meng Q. Sun B. Zhao B. Dang L. Zhong M. (2020). MeCas12a, a highly sensitive and specific system for COVID-19 detection. Adv. Sci. (Weinh). 7 (20), 2001300. 10.1002/advs.202001300 33042732 PMC7536916

[B52] Makarova K. S. Wolf Y. I. Alkhnbashi O. S. Costa F. Shah S. A. Saunders S. J. (2015). An updated evolutionary classification of CRISPR-Cas systems. Nat. Rev. Microbiol. 13 (11), 722–736. 10.1038/nrmicro3569 26411297 PMC5426118

[B53] Makarova K. S. Wolf Y. I. Iranzo J. Shmakov S. A. Alkhnbashi O. S. Brouns S. J. J. (2020). Evolutionary classification of CRISPR-cas systems: A burst of class 2 and derived variants. Nat. Rev. Microbiol. 18 (2), 67–83. 10.1038/s41579-019-0299-x 31857715 PMC8905525

[B54] Makarova K. S. Wolf Y. I. Koonin E. V. (2018). Classification and nomenclature of CRISPR-cas systems: where from here? Crispr J. 1 (5), 325–336. 10.1089/crispr.2018.0033 31021272 PMC6636873

[B55] Makarova K. S. Zhang F. Koonin E. V. (2017). SnapShot: class 1 CRISPR-cas systems. Cell 168 (5), 946. 10.1016/j.cell.2017.02.018 28235204

[B56] Myhrvold C. Freije C. A. Gootenberg J. S. Abudayyeh O. O. Metsky H. C. Durbin A. F. (2018). Field-deployable viral diagnostics using CRISPR-Cas13. Sci. (New York, NY) 360 (6387), 444–448. 10.1126/science.aas8836 PMC6197056 29700266

[B57] Najah S. Saulnier C. Pernodet J. L. Bury-Moné S. (2019). Design of a generic CRISPR-Cas9 approach using the same sgRNA to perform gene editing at distinct loci. BMC Biotechnol. 19 (1), 18. 10.1186/s12896-019-0509-7 30894153 PMC6425556

[B58] O'Connell M. R. (2019). Molecular mechanisms of RNA targeting by cas13-containing type VI CRISPR-cas systems. J. Mol. Biol. 431 (1), 66–87. 10.1016/j.jmb.2018.06.029 29940185

[B59] Pang B. Xu J. Liu Y. Peng H. Feng W. Cao Y. (2020). Isothermal amplification and ambient visualization in a single tube for the detection of SARS-CoV-2 using loop-mediated amplification and CRISPR technology. Anal. Chem. 92 (24), 16204–16212. 10.1021/acs.analchem.0c04047 33238709

[B60] Pardee K. Green A. A. Takahashi M. K. Braff D. Lambert G. Lee J. W. (2016). Rapid, low-cost detection of Zika virus using programmable biomolecular components. Cell 165 (5), 1255–1266. 10.1016/j.cell.2016.04.059 27160350

[B61] Patchsung M. Jantarug K. Pattama A. Aphicho K. Suraritdechachai S. Meesawat P. (2020). Clinical validation of a Cas13-based assay for the detection of SARS-CoV-2 RNA. Nat. Biomed. Eng. 4 (12), 1140–1149. 10.1038/s41551-020-00603-x 32848209

[B62] Pickar-Oliver A. Gersbach C. A. (2019). The next generation of CRISPR-Cas technologies and applications. Nat. Rev. Mol. Cell Biol. 20 (8), 490–507. 10.1038/s41580-019-0131-5 31147612 PMC7079207

[B63] Pinilla-Redondo R. Mayo-Muñoz D. Russel J. Garrett R. A. Randau L. Sørensen S. J. (2020). Type IV CRISPR-Cas systems are highly diverse and involved in competition between plasmids. Nucleic Acids Res. 48 (4), 2000–2012. 10.1093/nar/gkz1197 31879772 PMC7038947

[B64] Qian W. Huang J. Wang T. He X. Xu G. Li Y. (2021b). Visual detection of human metapneumovirus using CRISPR-Cas12a diagnostics. Virus Res. 305, 198568. 10.1016/j.virusres.2021.198568 34555442

[B65] Qian W. Huang J. Wang X. Wang T. Li Y. (2021a). CRISPR-Cas12a combined with reverse transcription recombinase polymerase amplification for sensitive and specific detection of human norovirus genotype GII.4. Virology 564, 26–32. 10.1016/j.virol.2021.09.008 34601182

[B66] Quan J. Langelier C. Kuchta A. Batson J. Teyssier N. Lyden A. (2019). Flash: A next-generation CRISPR diagnostic for multiplexed detection of antimicrobial resistance sequences. Nucleic Acids Res. 47 (14), e83. 10.1093/nar/gkz418 31114866 PMC6698650

[B67] Shmakov S. Abudayyeh O. O. Makarova K. S. Wolf Y. I. Gootenberg J. S. Semenova E. (2015). Discovery and functional characterization of diverse class 2 CRISPR-cas systems. Mol. Cell 60 (3), 385–397. 10.1016/j.molcel.2015.10.008 26593719 PMC4660269

[B68] Shmakov S. Smargon A. Scott D. Cox D. Pyzocha N. Yan W. (2017). Diversity and evolution of class 2 CRISPR-Cas systems. Nat. Rev. Microbiol. 15 (3), 169–182. 10.1038/nrmicro.2016.184 28111461 PMC5851899

[B69] Sternberg S. H. Redding S. Jinek M. Greene E. C. Doudna J. A. (2014). DNA interrogation by the CRISPR RNA-guided endonuclease Cas9. Nature 507 (7490), 62–67. 10.1038/nature13011 24476820 PMC4106473

[B70] Stower H. (2018). CRISPR-based diagnostics. Nat. Med. 24 (6), 702. 10.1038/s41591-018-0073-z 29875459

[B71] Swarts D. C. Jinek M. (2019). Mechanistic insights into the cis- and trans-acting DNase activities of Cas12a. Mol. Cell 73 (3), 589–600. 10.1016/j.molcel.2018.11.021 30639240 PMC6858279

[B72] Ting W. Yong L. Huan-Huan S. Yin B. C. Ye B. C. (2019). An RNA-guided Cas9 nickase-based method for universal isothermal DNA amplification. Angewandte Chemie Int. ed Engl. 58 (16), 5382–5386. 10.1002/anie.201901292 30773764

[B73] Wang B. Wang R. Wang D. Wu J. Li J. Wang J. (2019). Cas12aVDet: A CRISPR/cas12a-based platform for rapid and visual nucleic acid detection. Anal. Chem. 91 (19), 12156–12161. 10.1021/acs.analchem.9b01526 31460749

[B74] Weissleder R. Lee H. Ko J. Pittet M. J. (2020). COVID-19 diagnostics in context. Sci. Transl. Med. 12 (546), eabc1931. 10.1126/scitranslmed.abc1931 32493791

[B75] Xin C. Yin J. Yuan S. Ou L. Liu M. Zhang W. (2022). Comprehensive assessment of miniature CRISPR-Cas12f nucleases for gene disruption. Nat. Commun. 13 (1), 5623. 10.1038/s41467-022-33346-1 36153319 PMC9509373

[B76] Yang H. Chen J. Yang S. Zhang T. Xia X. Zhang K. (2021b). CRISPR/Cas14a-Based isothermal amplification for profiling plant MicroRNAs. Anal. Chem. 93 (37), 12602–12608. 10.1021/acs.analchem.1c02137 34506121

[B77] Yang S. Rothman R. E. (2004). PCR-Based diagnostics for infectious diseases: uses, limitations, and future applications in acute-care settings. Lancet Infect. Dis. 4 (6), 337–348. 10.1016/S1473-3099(04)01044-8 15172342 PMC7106425

[B78] Yang Y. Liu J. Zhou X. (2021a). A CRISPR-based and post-amplification coupled SARS-CoV-2 detection with a portable evanescent wave biosensor. Biosens. Bioelectron. 190, 113418. 10.1016/j.bios.2021.113418 34119838 PMC8182983

[B79] Yao R. Liu D. Jia X. Yuan Z. Liu W. Xiao Y. (2018). CRISPR-Cas9/Cas12a biotechnology and application in bacteria. Synthetic Syst. Biotechnol. 3, 135–149. 10.1016/j.synbio.2018.09.004 PMC6190536 30345399

[B80] Yoshimi K. Takeshita K. Yamayoshi S. Shibumura S. Yamauchi Y. Yamamoto M. (2022). CRISPR-Cas3-based diagnostics for SARS-CoV-2 and influenza virus. iScience 25 (2), 103830. 10.1016/j.isci.2022.103830 35128347 PMC8801231

[B81] Yuan B. Yuan C. Li L. Long M. Chen Z. (2022). Application of the CRISPR/cas system in pathogen detection: A review. Molecules 27 (20), 6999. 10.3390/molecules27206999 36296588 PMC9610700

[B82] Zeng R. Xu J. Lu L. Lin Q. Huang X. Huang L. (2022). Photoelectrochemical bioanalysis of microRNA on yolk-in-shell Au@CdS based on the catalytic hairpin assembly-mediated CRISPR-Cas12a system. Chem. Commun. (Camb). 58 (54), 7562–7565. 10.1039/d2cc02821b 35708478

[B83] Zetsche B. Gootenberg J. S. Abudayyeh O. O. Slaymaker I. M. Makarova K. S. Essletzbichler P. (2015). Cpf1 is a single RNA-guided endonuclease of a class 2 CRISPR-Cas system. Cell 163 (3), 759–771. 10.1016/j.cell.2015.09.038 26422227 PMC4638220

[B84] Zhang W. S. Pan J. Li F. Zhu M. Xu M. Zhu H. (2021). Reverse transcription recombinase polymerase amplification coupled with CRISPR-cas12a for facile and highly sensitive colorimetric SARS-CoV-2 detection. Anal. Chem. 93 (8), 4126–4133. 10.1021/acs.analchem.1c00013 33570401

[B85] Zhang X. An X. (2022). Adaptation by type III CRISPR-cas systems: breakthrough findings and open questions. Front. Microbiol. 13, 876174. 10.3389/fmicb.2022.876174 35495695 PMC9048733

[B86] Zhang Y. Wang Y. Xu L. Lou C. Ouyang Q. Qian L. (2022). Paired dCas9 design as a nucleic acid detection platform for pathogenic strains. Methods (San Diego, Calif. 203, 70–77. 10.1016/j.ymeth.2021.06.003 34090973

[B87] Zhao H. Sheng G. Wang J. Wang M. Bunkoczi G. Gong W. (2014). Crystal structure of the RNA-guided immune surveillance Cascade complex in *Escherichia coli* . Nature 515 (7525), 147–150. 10.1038/nature13733 25118175

[B88] Zheng Y. Li J. Wang B. Han J. Hao Y. Wang S. (2020). Endogenous type I CRISPR-cas: from foreign DNA defense to prokaryotic engineering. Front. Bioeng. Biotechnol. 8, 62. 10.3389/fbioe.2020.00062 32195227 PMC7064716

